# Astragalus–Scorpion Drug Pair Inhibits the Development of Prostate Cancer by Regulating GDPD4-2/PI3K/AKT/mTOR Pathway and Autophagy

**DOI:** 10.3389/fphar.2022.895696

**Published:** 2022-06-29

**Authors:** Xujun You, Yongrong Wu, Qixin Li, Wen Sheng, Qing Zhou, Wei Fu

**Affiliations:** ^1^ Graduate School of Hunan University of Chinese Medicine, Changsha, China; ^2^ Department of Andrology, Shenzhen Bao’an Traditional Chinese Medicine Hospital, Guangzhou University of Chinese Medicine, Shenzhen, China; ^3^ College of Integrated Traditional Chinese and Western Medicine, Hunan University of Chinese Medicine, Changsha, China; ^4^ Andrology Laboratory, Hunan University of Chinese Medicine, Changsha, China; ^5^ Department of Andrology, The First Affiliated Hospital of Hunan University of Chinese Medicine, Changsha, China

**Keywords:** Astragalus–Scorpio, prostate cancer, PI3K/AKT, Astragaloside IV, polypeptide extract from scorpion venom, autophagy

## Abstract

**Objective:** Prostate cancer (PCa) is an epithelial malignancy of the prostate that currently lacks effective treatment. Traditional Chinese medicine (TCM) can play an anticancer role through regulating the immune system, anti-tumor angiogenesis, regulating tumor cell apoptosis, autophagy dysfunction, and other mechanisms. This study attempted to explore the active ingredients and potential mechanism of action of the Astragalus–Scorpion (A–S) drug pair in PCa, in order to provide new insights into the treatment of PCa.

**Methods:** Network pharmacology was used to analyze the A–S drug pair and PCa targets. Bioinformatics analysis was used to analyze the LncRNAs with significant differences in PCa. The expression of LC3 protein was detected by immunofluorescence. CCK8 was used to detect cell proliferation. The expressions of GDPD4-2, AC144450.1, LINC01513, AC004009.2, AL096869.1, AP005210.1, and BX119924.1 were detected by RT-qPCR. The expression of the PI3K/AKT/mTOR pathway and autophagy-related proteins were detected by western blot. LC-MS/MS was used to identify the active components of Astragalus and Scorpion.

**Results:** A–S drug pair and PCa have a total of 163 targets, which were mainly related to the prostate cancer and PI3K/AKT pathways. A–S drug pair inhibited the formation of PCa, promoted the expression of LC3Ⅱ and Beclin1 proteins, and inhibited the expression of P62 and PI3K–AKT pathway proteins in PCa mice. Astragaloside IV and polypeptide extract from scorpion venom (PESV) were identified as the main active components of the A–S drug pair. GDPD4-2 was involved in the treatment of PCa by Astragaloside IV-PESV. Silencing GDPD4-2 reversed the therapeutic effects of Astragaloside IV-PESV by regulating the PI3K/AKT/mTOR pathway.

**Conclusion:** Astragaloside IV-PESV is the main active components of A–S drug pair treated PCa by regulating the GDPD4-2/PI3K–AKT/mTOR pathway and autophagy.

## Introduction

Prostate cancer (PCa) is one of the most common malignant tumors in men, with high morbidity and mortality worldwide (de Bono et al., 2020). In 2020, there are estimated to be more than 1,414,000 new cases of PCa worldwide (Gandaglia et al., 2021). PCa causes deaths, mostly due to incurable metastatic diseases (Buckup et al., 2021; Peng et al., 2021). Autophagy related genes (ARGs) play an important role in many biological processes of PCa (Hu et al., 2020). Within tumor masses, autophagy promotes cell survival by increasing the tolerance of cancer cells to different cellular stresses, such as hypoxia, starvation, or those triggered by chemotherapy drugs (Cristofani et al., 2018). Of note, androgen deprivation therapy, taxane-based chemotherapy, targeted kinase inhibition, and nutritional restriction all cause significant cellular distress and subsequent autophagy (Farrow et al., 2014). Preclinical studies have shown that the pharmacological inhibition of autophagy (e.g., metformin) can enhance the cell-killing effects of cancer drugs (Ahn et al., 2020; Chen C. et al., 2021). Therefore, exploring the pharmacology of autophagy associated with PCa may contribute to the development of new therapeutic strategies.

Network pharmacology and molecular docking techniques suggested that Astragalus–Scorpion (A–S) drug pair has the potential to treat PCa (Wu et al., 2021). Astragalus polysaccharides, a traditional Chinese medicine (TCM), have been proven to inhibit tumor genesis and lipid metabolism through the miR-138-5p/SIRT1/SREBP1 pathway in PCa (Guo et al., 2020). Astragalus membranaceus, Angelica gigas, and Trichosanthes Kirilowii Maximowicz (1:1:1) extract induced apoptosis of PCa cells by inhibiting ERK2-mediated signaling (Choi et al., 2016). Scorpion is a TCM for animals with complex main active ingredients. San’ao decoction with scorpion has been shown to relieve asthma (Wang P. et al., 2021). It is well known that scorpion venom is the leading cause of human poisoning and even death by scorpion sting (So et al., 2021). Reports in recent years indicate that scorpion venom may be the main active ingredient in scorpion that exerts anticancer effects. For example, Androctonus amoreuxi scorpion venom significantly has cytotoxic and anti-proliferative effects on PCa cells (Akef et al., 2017). Polypeptide extracted from scorpion venom (PESV) induces the growth inhibition of PCa cells (Zhang et al., 2009). However, the mechanism of action of the A–S drug pair in the treatment of PCa remains unclear.

Phosphatidylinositol 3-kinase (PI3K)/AKT pathway was associated with the development of PCa (Chen et al., 2016b). PI3K can be used as a useful biomarker for the early diagnosis and prognosis of biochemical recurrence of PCa after radical prostatectomy (Torrealba et al., 2020). Recent findings suggested that the complex crosstalk between the PI3K/AKT/mTOR pathway and multiple interacting cellular signaling cascades could further promote the progression of PCa and influenced the sensitivity of PCa cells to PI3K/AKT/mTOR-targeted therapies being explored clinically (Shorning et al., 2020). Studies have demonstrated that salamycin-induced apoptosis of PCa cells by regulating the PI3K/AKT/mTOR signaling pathway, which was associated with ROS-mediated autophagy (Kim et al., 2017). In addition, the inhibition of the PI3K/AKT/mTOR/70S6K pathway could promote autophagy in LNCaP cells (Butler et al., 2017; Dai et al., 2021).

It is well known that the PI3K/AKT/mTOR pathway, as a canonical pathway of autophagy, is involved in PCa (Kim et al., 2017). The long non-coding RNAs (lncRNAs) may affect PCa development by regulating the PI3K pathway (Chen et al., 2020; Liu et al., 2020; Peng et al., 2021; Sun et al., 2021; Yang et al., 2021). Among them, SNHG1 and ADAMTS9-AS1 are closely related to autophagy regulation (Chen et al., 2020; Yang et al., 2021). In addition, the TCM, Jixuepaidu Tang-1, and Astragaloside IV have also been confirmed to be involved in the process of liver cancer and kidney injury by regulating ATB (Li et al., 2018) and LOC498759 (Jin et al., 2019), respectively. It was known that the treatment of flavanol glycoside icaridin, the active ingredient of TCM, could inhibit the proliferation and migration of human PCa cells and enhance autophagy by regulating the PI3K/AKT/mTOR signaling pathway (Li S. et al., 2020). However, the effects of the A–S drug pair on the PI3K pathway, autophagy, and lncRNAs have never been studied in PCa. Therefore, this study intends to explore the active ingredients, predicted targets, and possible molecular mechanism of the A–S drug pair through network pharmacology (Hopkins, 2008) and explore the internal molecular mechanism of the A–S drug pair in the treatment of PCa, so as to provide a theoretical basis for the clinical treatment of PCa with TCM.

## Materials and Methods

### Network Pharmacology Analysis

The A–S drug pair active ingredients were retrieved through TCMSP and TCMID databases. The component screening conditions were OB ≥ 30% and DL ≥ 0.18. “Prostatic cancer” as keywords in GeneCards database (https://www.genecards.org/), NCBI databases (https://www.ncbi.nlm.nih.gov/), and OMIM database (https://www.omim.org/) were retrieved for human gene. The ImageGP platform was used to match and overlap the targets corresponding to the active ingredients in the A–S drug pair with those of PCa, and the Venn diagram was drawn to obtain the key targets of the A–S drug pair active ingredients in the treatment of PCa. The PPI network was constructed by inputting the common targets of drug diseases into the String database (https://string-db.org/cgi/input.pl), and the species was set as “*Homo sapiens*” to obtain the PPI network. PPI network was imported into Cystoscape 3.8.0 (Doncheva et al., 2019), topology analysis was conducted by NetworkAnalyzer tool, degree sequencing, and genes with scores greater than average were selected as key targets. KEGG pathway enrichment analysis was performed on the common targets of drug diseases, and the items with a corrected *p* value <0.05 were screened by using the String database. Using R 4.0.3, after installing and referencing the clusterProfiler package, the bar and bubble charts are drawn. The chemical structures of various screened active ingredient monomers were analyzed (https://pubchem.ncbi.nlm.nih.gov/) (Kim et al., 2021).

### A Mouse Model of PCa

All animal experiments were approved by the Experimental Animal Ethics Committee of Guangzhou University of Chinese Medicine (No. 20210224026). In this study, 110 male BABL/c nude mice (4–6 weeks) were purchased from Hunan Slacker Jingda Laboratory Animal Co., Ltd. Model construction was described as follows (Li J. et al., 2020; Mehra et al., 2021): 2 × 10^6^ cells were resuspended in 100 µL serum-free RPMI containing 50% Matrigel (356234, ThermoFisher) and injected subcutaneously into the left side of an athymic mouse. The mice were divided into groups 1 week after injection.

### Sources of A–S Drug Pair

Astragalus was purchased from China Resources Sanjiu Pharmaceutical Co., Ltd. (2101006c). Scorpion was obtained from Yifang Pharmaceutical, Guangdong, China (1013451). Astragalus and scorpion granules were dissolved in distilled water for subsequent experiments.

### Liquid Chromatograph-Mass Spectrometer Analysis of Astragalus

A Shimadzu Prominence UPLC system (Nexera UHPLC LC-30A, Kyoto, Japan) coupled with an AB SCIEX Triple TOF 5600 + system (AB Sciex, Singapore) equipped with an ESI source was used to analyze Astragalus. T3 column (2.1 × 100 mm with 1.7 μm particle size, Waters, Milford, MA, United States) was utilized to separate samples (3 μL). The column temperature was maintained at 40°C. Mobile phases include 0.1% formate in H_2_O (mobile phase A) and acetonitrile (mobile phase B). The mass spectrometer has both positive and negative ion modes. The detailed instrument parameters are as follows. The source temperature was 550°C. The ion source gas was 1 and 2 55 psi. The curtain gas was 35 psi. The ion spray voltage float was 5.5 kV in a positive mode and −4.5 kV in a negative mode. The accumulation time for the full scan was 150 ms, and the accumulation time for each IDA experiment was 45 ms. The mass range was set to m/z 60 to m/z 1,250, and the collision energy was set to 30 eV or −30 eV. Peaks for compounds with intensities greater than 100 c.p.s. were selected for further analysis after summing the signals from 10 rounds of IDA scans.

### Component Analysis of Scorpion

The scorpion particles were derived from the whole body of *Buthus martensii Karsch* (BMK), a scorpionidae animal, including scorpion venom. PESV is a polypeptide extracted from scorpion venom. Scorpion particles (0.1 g) were dissolved in 1 ml RIPA lysate and then vortexed for 20 min. The supernatant was transferred to the new EP tube after ultrasonic extraction for 15 min and centrifugation for 5 min. The 20 μL supernatant was taken and run on SDS-PAGE gel for 2 h. The gel was stained with Coomassie bright blue solution for 2 h and decolorized overnight. The albumin glue was cut off with a disposable surgical blade and transferred to a new EP tube. The dyed strips were rinsed three times by ddH_2_O and added into the decolorizing solution. After decolorization, the rubber strip was washed 3–5 times until completely transparent. The rubber strip was washed with 50% acetonitrile and 100% acetonitrile to dehydrate the rubber until the rubber block became white. To the rubber strip was added 50 μL DTT solution (10 mM) and reduced for 30 min in a water bath at 56°C. When the temperature drops to room temperature, 50 mM IAA solution with equal volume was added to avoid light alkylation for 15 min. The rubber strip was washed with 50% acetonitrile and 100% acetonitrile twice in order to dehydrate the rubber until the rubber block became white. To the rubber strip was added 15–20 μL proteome-grade trypsin (0.01 μg/μL) and put on ice to absorb and become transparent. To the rubber strip was added 30–40 μL NH_4_HCO_3_ solution (50 mM) containing 10% ACN to cover it. After digestion overnight in the water bath at 37°C, the supernatant was transferred to another new EP tube. 100 µL of extraction solution (67% acetonitrile, containing 2% formic acid) was added to the remaining colloidal blocks and held for 30 min at 37°C. After centrifugation and drying, the enzyme-cut polypeptide samples were re-dissolved in a nano-LC mobile phase A (0.1% formic acid/water), bottled, and sampled for LC/MS analysis. LC/MS analysis was performed by an Easy nLC 1200 Nano Liter liquid phase system (ThermoFisher, United States) and a ThermoFisher Q Exactive System (ThermoFisher, United States) combined Nano liter spray Nano Flex ion source (ThermoFisher, United States). The original RAW atlas files collected by mass spectrometry were processed and retrieved using PEAKS Studio 8.5 (Bioinformatics Solutions Inc. Waterloo, Canada) software. The database was the target protein database downloaded by Uniprot. The retrieval parameters were set as follows: trypsin enzymolysis, the mass tolerance of primary mass spectrometry was 10 ppm, and the secondary mass spectrometry was 0.05 Da.

### Animal Experiment and Grouping

BABL/c nude male PCa mice constructed from LNCap cells were randomly divided into seven groups. The group was set as the model group (PCa), a low dose of the A–S drug pair group (A–S–L, 1.17 g/kg/d A and 0.39 g/kg/d S), a middle dose of the A–S drug pair group (A–S–M, 2.54 g/kg/d A and 0.585 g/kg/d S), a high dose of the A–S drug pair group (A–S–H, 3.9 g/kg/d A and 0.78 g/kg/d S), Astragalus group (A, 3.9 g/kg/d), scorpion group (S, 0.78 g/kg/d), and docetaxel (D107320, Aladdin) group (10 mg/kg, once a week, intraperitoneal injection). The A–S drug pair was administered orally by gavage, and the control group was given the same volume of distilled water. Tumor growth and metastasis in mice were monitored every 7 days using small animal *in vivo* imaging system (IVIS).

BABL/c nude male PCa mice constructed from sh-GDPD4-2 stable LNCaP cells were randomly divided into four groups. The group was set as the sh-NC group, sh-GDPD4-2 group, Astragaloside IV-PESV group, and Astragaloside IV-PESV + sh-GDPD4-2 group. Astragaloside IV (84687-43-4, Aladdin) was given 40 mg/kg by gavage (Nanding Wang et al., 2020). PESV was an intraperitoneal injection of 1.2 mg/kg. PESV was obtained from the Chinese Medicine Pharmacy of the First Affiliated Hospital of Hunan University of Chinese Medicine. The experiment lasted for 4 weeks, and drug administration began 1 week after implantation. Changes in the tumor volume and mortality of mice were detected by the IVIS system.

### Cell Experiment and Grouping

Human normal prostate epithelial cells RWPE-1 cells (iCell-h286, iCell) and human prostate cancer cell line LNCaP (CL-0143, Procell) were purchased from iCell and Procell, respectively. The LNCaP cells were randomly divided into LNCaP group (control), LNCaP + Astragaloside IV group (Astragaloside IV, 10μM, 24 h), LNCaP + PESV group (PESV, 40 mg/ml, 24 h), LNCaP + Astragaloside IV-PESV group (Astragaloside IV-PESV), and LNCaP + rapamycin group (rapamycin, 30 nM, 24 h). The sh-GDPD4-2 stable LNCaP cells were randomly divided into negative control group (NC), sh-GDPD4-2 group (sh-GDPD4-2), NC + Astragaloside IV-PESV group (Astragaloside IV-PESV), and sh-GDPD4-2 + Astragaloside IV-PESV group (sh-GDPD4-2 + Astragaloside IV-PESV).

### Bioinformatics Analysis

The original data set of GSE155056 was downloaded in the GEO database, converted into expression matrix, and the grouping information was obtained. The expression matrix was processed with the R language Limma package, and the expression difference was analyzed. According to the criteria (*p* value <0.05, | log_2_FC | > 2.0), statistically significant genes were screened, and the map of volcanic was masked. The clustering heatmap showed the expression patterns of differentially expressed lncRNAs in the data set.

### Immunofluorescence

The cells were implanted with slides and fixed with 4% paraformaldehyde for 30 min. Triton of 0.3% was added and permeated at 37°C for 30 min, and 5% BSA was sealed at 37°C for 60 min, and the antibody was incubated after washing with PBS. The tumor tissues of mice were fixed, dehydrated, and paraffin-embedded. The tissue was cut into 5 μm slices. Sections were dewaxed to water using gradient alcohol (75–100%). Sections were immersed in 0.01 M citrate buffer (pH6.0) for the thermal repair of antigen. Then, 1% periodate acid was added at room temperature for 10 min to inactivate endogenous enzymes. Sections were dropped with appropriately diluted anti-LC3 (14600-1-AP, 1:100, Proteintech, United States) at 4°C overnight. Sections were added to 50–100 μL anti-HRP polymer and incubated at 37°C for 30 min. DAB working solution was used for incubation, hematoxylin restaining, and gradient alcohol dehydration. Neutral gum was sealed and observed under a microscope (BA410T, Motic).

### Cell Counting Kit-8

The cells were digested and inoculated into 96-well plates at a density of 5×10^3^ cells/well, 100 μL per well. Then, 10 μL CCK8 (NU679, DOJINDO) solution was added to each well. Cells were incubated at 37°C with 5% CO_2_ for 4 h. A Bio-tek microplate analyzer (mb-530, huisong) was used to analyze the absorbance at 450 nm.

### Quantitative Reverse Transcription PCR

Total RNA was extracted by TRIzol reagent (15596026, Thermo). cDNA was synthesized using an mRNA reverse transcription kit (CW2569, CWBIO). The sequences of target genes were searched on NCBI, and primer 5 software was used to design primers. The expression levels of target genes were analyzed by an UltraSYBR Mixture (CW2601, CWBIO) and 2^−ΔΔCT^. Primer sequences are shown in Table 1.

**TABLE 1:** Primer sequences.

Gene	Primer sequences	Length (bp)
H-GDPD4-2	F ACC​AGG​ATC​CCA​TTC​CTA​CCA	80
	R GGT​CAC​TGT​CCA​CGC​ACA​AA	
H-AC144450.1	F GTG​GTG​TGA​CGA​CAT​CCT​GT	163
	R GGT​AGC​TCT​GCG​GTC​AAT​CA	
H-LINC01513	F GGA​GAC​ACC​ACC​TCT​TTG​CT	75
	R TGA​CTC​TCC​TCT​TGT​TCC​AGA​T	
H-AC004009.2	F GGT​CCT​GAC​ACG​GGC​ATT​C	178
	R GGG​CAA​AAG​CAA​CCT​TTC​AG	
H-AL096869.1	F CAC​TGC​TCT​GGA​CCC​TTG​AG	149
	R CCC​TGT​GTA​GGC​ATG​TCC​AG	
H-AP005210.1	F ATC​TCC​AGC​CAA​TCA​GTC​ACC	173
	R AGA​CAT​GAA​GAA​AAT​CCG​CCA​T	
H-BX119924.1	F GAA​CCC​GTC​TGC​GTT​TCT​CC	85
	R GCC​ACA​AAG​TAC​AAA​GCG​AGG	
H-GAPDH	F ACA​GCC​TCA​AGA​TCA​TCA​GC	104
	R GGT​CAT​GAG​TCC​TTC​CAC​GAT	

### Western Blot

RIPA lysate was used to lysate cells or mouse tumor tissues to extract total protein. The concentration of the extracted protein was determined by the BCA protein quantitative kit. Protein samples were separated by sodium dodecyl sulfate-polyacrylamide gel electrophoresis (SDS-PAGE). The isolated proteins were transferred to a polyvinylidene fluoride (PVDF) membrane activated by methanol and sealed by 5% skim milk and dried at room temperature for at least 1 h. Then, they were incubated with anti-LC3B (14600-1-AP, 1:1000, Proteintech, United States), anti-Beclin1 (ab210498, 1:1000, Abcam, United Kingdom), anti-P62 (18420-1-AP, 1:5000, Proteintech, United States), anti-p-PI3K (ab191606, 1:1000, Abcam, United Kingdom), anti-PI3K (ab182651, 1:1000, Abcam, United Kingdom), anti-p-AKT (66444-1-Ig, 1:2000, ProteinTech, United States), anti-AKT (10176-2-AP, 1:2000, Proteintech, United States), anti-p-mTOR (ab109268, 1: 2000, Abcam, United Kingdom), anti-mTOR (ab2732, 1:500, Abcam, United Kingdom), anti-β-actin (66009-1-Ig, 1:5000, Proteintech, United States) overnight at 4°C. It was then incubated with secondary anti-IgG (SA00001-1, 1:5000, Proteintech, United States) and anti-IgG (SA00001-2, 1:6000, Proteintech, United States) at 37°C for 90 min. Visualization was performed using chemiluminescence, and imaging analysis was performed using software (GE Healthcare, Life Sciences, United States).

### Statistics and Analysis

Graphpad prism 8 was used for statistical analysis of the research data. The measurement data were expressed as mean ± SD. First, the test of normality and homogeneity of variance were carried out. The test conforms to the normal distribution and homogeneity of variance. The non-paired *t*-test was used between groups; the one-way ANOVA or ANOVA of repeated measurement data was used for multi-group comparison, and Tukey’s post test was carried out. *p* < 0.05 indicated that the difference was statistically significant.

## Results

### Network Pharmacologic Analysis of A–S Drug Pair in Treating PCa

**FIGURE 1:**
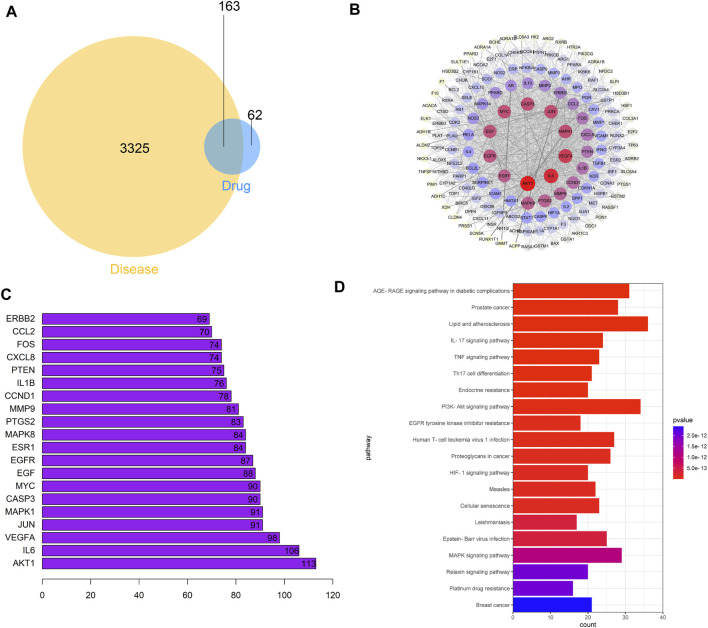
Potential effect mechanism of A–S for treating PCa based on network pharmacology analysis. **(A)** Venn diagrams of common targets in PCa and A–S. **(B)** Construction of the PPI network in A–S in treating PCa by using the STRING database. **(C)** The top 20 core gene visualization was obtained by using R software according to the relevance number of nodes. **(D)** KEGG pathway analysis of core targets and column plot for top 20 pathways.

Venn diagram showed that the A–S drug pair and PCa had 163 targets (Figure 1A). There were 163 nodes in PPI network, and the average degree value was 35 (Figure 1B). Key target analysis showed that AKT1 was the target gene with the highest degree value (Figure 1C). KEGG functional enrichment showed that a total of 165 signaling pathways were enriched, among which the prostate cancer and PI3K–AKT signaling pathway are significantly enriched (Figure 1D). The chemical structures of various screened active ingredient monomers in the A–S drug pair are shown in Supplementary Figure S1. These results suggested that the A–S drug pair may play a pharmacological role in the treatment of PCa through the PI3K–AKT signaling pathway.

### A–S Drug Pair Inhibited the Development of PCa

**FIGURE 2:**
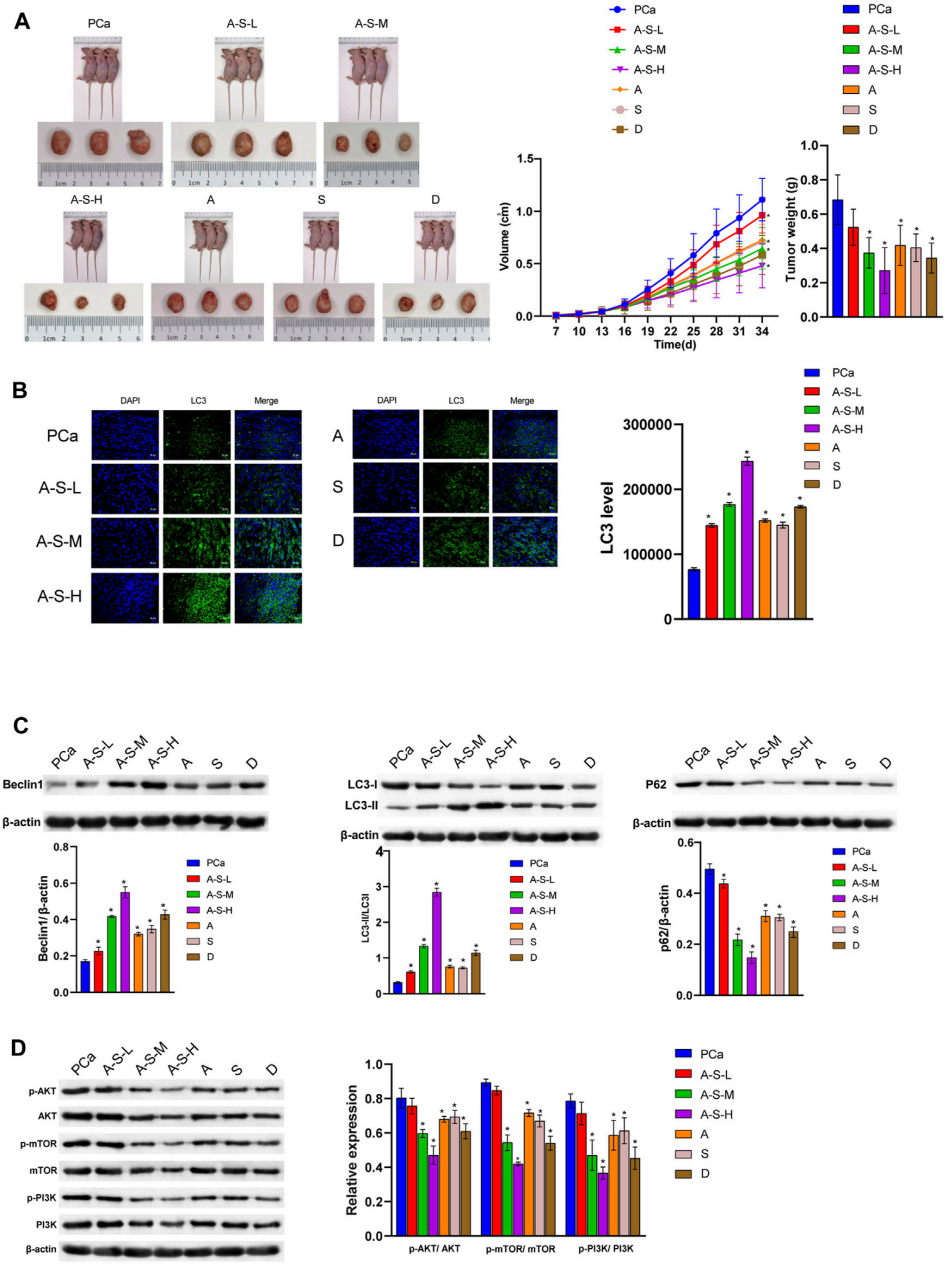
A –S drug pair inhibited PCa development by inhibiting the PI3K–AKT/mTOR pathway. **(A)** LNCaP cells were injected subcutaneously into BABL/c nude mice (*n* = 6), and the tumor volume and tumor weight were analyzed. **(B)** LC3 and DAPI immunofluorescence staining were performed to detect the LC3 expression. **(C)** The LC3, Beclin1, and P62 expression were determined by western blot. **(D)**The expression of the PI3K/AKT/mTOR pathway protein was analyzed by western blot. *p < 0.05 vs. control group.

The A–S drug pair extract significantly inhibited the formation of PCa, and the inhibition effect was better in the A–S–H group, as well as in the tumor volume and tumor weight (Figure 2A). In addition, the A–S–H drug pair extract was found to promote LC3Ⅱ and Beclin1 protein expression in PCa tissues (Figures 2B,C). Compared with the PCa group, the A–S–H drug pair extract significantly inhibited the expression of P62 protein in PCa mice (Figure 2C). Compared with the PCa group, the A–S–H group significantly inhibited the expression of p-PI3K, p-AKT, and p-mTOR proteins (Figure 2D). These results suggested that the A–S drug pair extract inhibited tumor formation in PCa mice, which may be related to the PI3K/AKT pathway and autophagy.

### Astragaloside IV and PESV as the Main Active Components of A–S Drug Pair Inhibited the Proliferation of LNCaP

**FIGURE 3:**
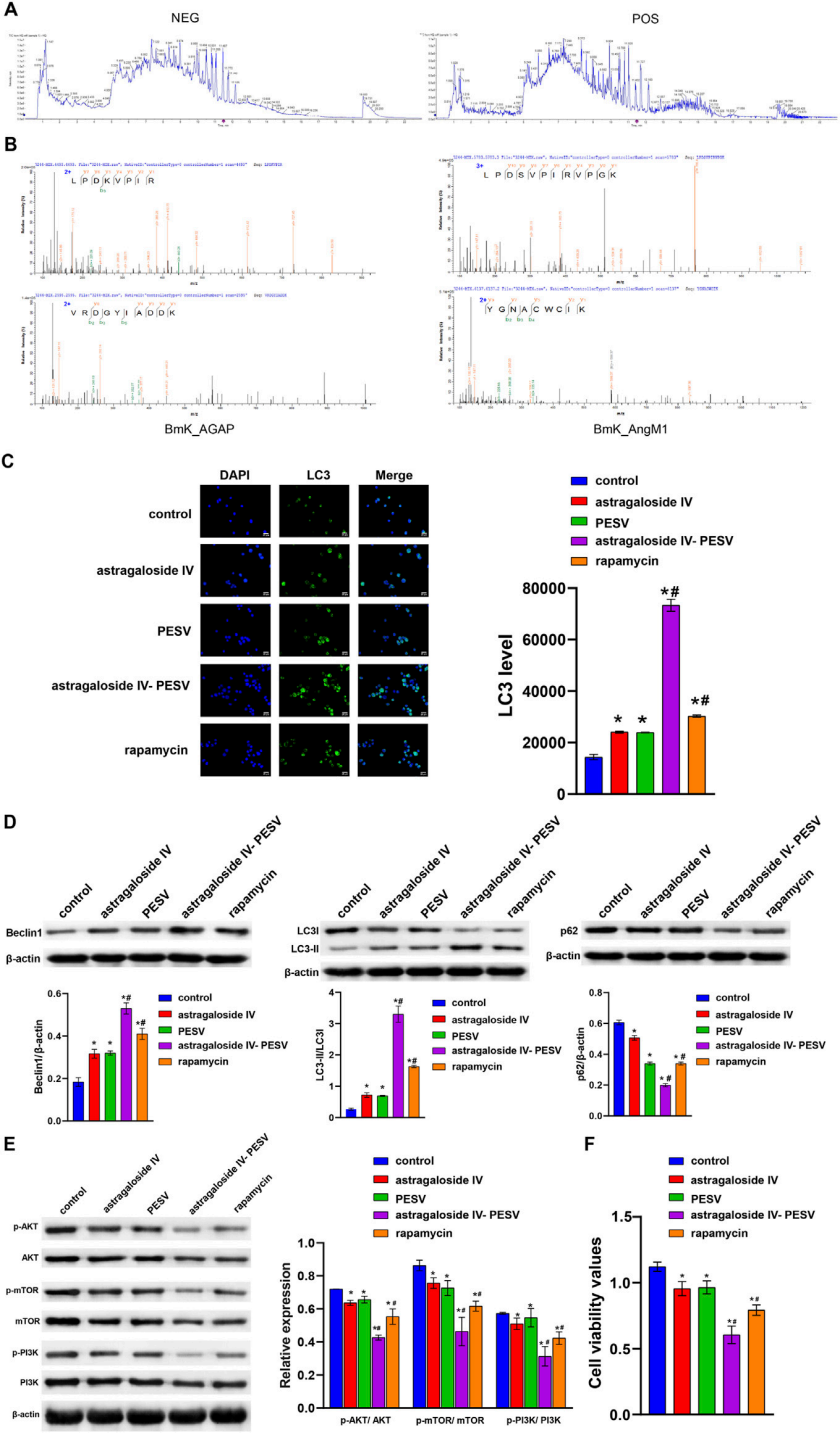
The main bio-active components in the herb pair A–S suppressed LNCaP cell proliferation and migration through the PI3K/AKT/mTOR pathway. **(A)** LC–MS/MS chromatogram of the bio-active components in Astragalus. **(B)** The LC-MS/MS analysis of PESV in scorpion. **(C)** LC3 and DAPI immunofluorescence staining were performed to detect autophagy. **(D)** The LC3, Beclin1, and P62 expression were determined by western blot. **(E)** The protein expression of the PI3K/AKT/mTOR signaling pathway. **(F)** Cell activity was determined by the CCK8 assay. *p < 0.05 vs. control group, #*p* < 0.05 vs. Astragaloside IV or PESV groups.

The positive and negative profiles of the Astragalus showed that Astragaloside IV was one of the main active components (Figure 3A). In order to identify whether the scorpion granules contain PESV, the scorpion granules were subjected to SDS-PAGE electrophoresis gel and enzymatic hydrolysis to separate the peptides and finally analyzed by LC-MS/MS. The results showed that the scorpion peptides Bmk_AGAP and BmK_AngM1 (unique active component present in the PESV extract) existed in the whole scorpion, which might be the key substance in the anticancer effect of the scorpion (Figure 3B). Compared with the control group, the LC3 expression was significantly increased in the Astragaloside IV and PESV groups (Figure 3C). Astragaloside IV-PESV significantly promoted LC3 and Beclin1 protein expression in LNCaP compared to Astragaloside IV or PESV groups (Figures 3C,D). Astragaloside IV-PESV inhibited the expression of P62, p-PI3K, p-AKT, and p-mTOR in LNCaP (Figures 3D,E). In addition, compared with Astragaloside IV or PESV groups, Astragaloside IV-PESV significantly inhibited the proliferation of LNCaP (Figure 3F). These results suggested that Astragaloside IV-PESV, as the main active components of the A–S drug pair, inhibited LNCaP proliferation through PI3K/AKT and autophagy pathways.

### Differential LncRNA Expression of PCa Treated With Astragaloside IV-PESV

**FIGURE 4:**
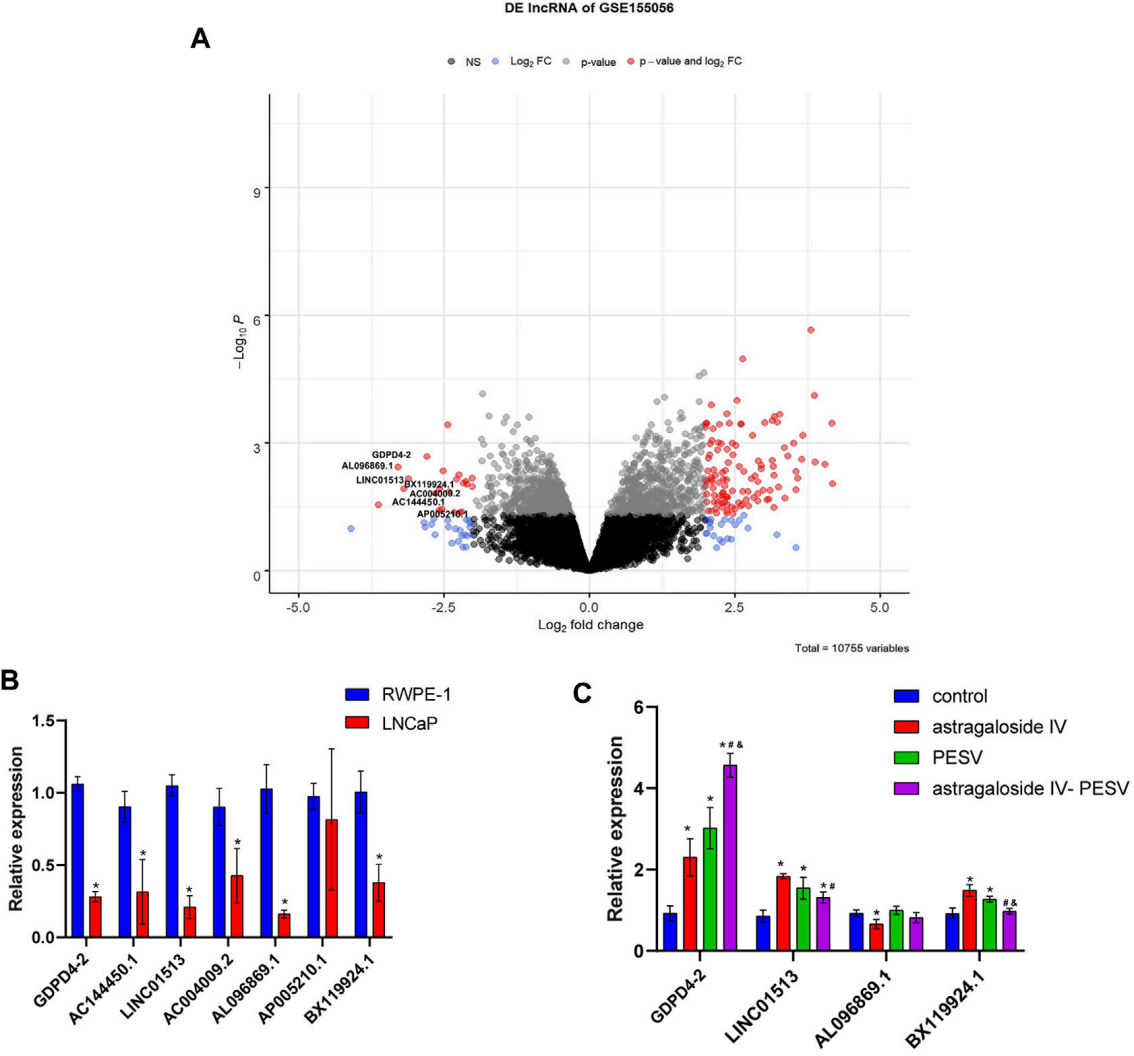
In PCa tissues and LNCaP cells, GDPD4-2 expression was decreased compared to non-tumorigenic prostate epithelial cells but increased following treatment with the herb pair Astragalus IV and PESV. **(A)** Volcano plot showed the lncRNA expression. **(B)** The differential lncRNA expression in RWPE-1 and LNCaP cells, *p < 0.05 vs. RWPE-1 group. **(C)** The differential lncRNA expression was analyzed by RT-qPCR in LNCaP cells after treatment with Astragaloside IV-PESV. *p < 0.05 vs. control group, #*p* < 0.05 vs. Astragaloside IV group, &*p* < 0.05 vs. PESV group.

The volcano plot showed that GDPD4-2, AC144450.1, LINC01513, AC004009.2, AL096869.1, AP005210.1, and BX119924.1 were significantly underexpressed (Figure 4A). Compared with the RWPE-1 cell, the GDPD4-2, AC144450.1, LINC01513, AC004009.2, AL096869.1, AP005210.1, and BX119924.1 were significantly decreased in LNCaP cell (Figure 4B). Compared with the control group, the expression of GDPD4-2 was significantly up-regulated in the Astragaloside IV-PESV group (Figure 4C). These results demonstrated that GDPD4-2 was involved in the treatment of PCa by Astragaloside IV-PESV.

### Astragaloside IV-PESV Regulated the PI3K/AKT/mTOR Signaling Pathway Through GDPD4-2

**FIGURE 5:**
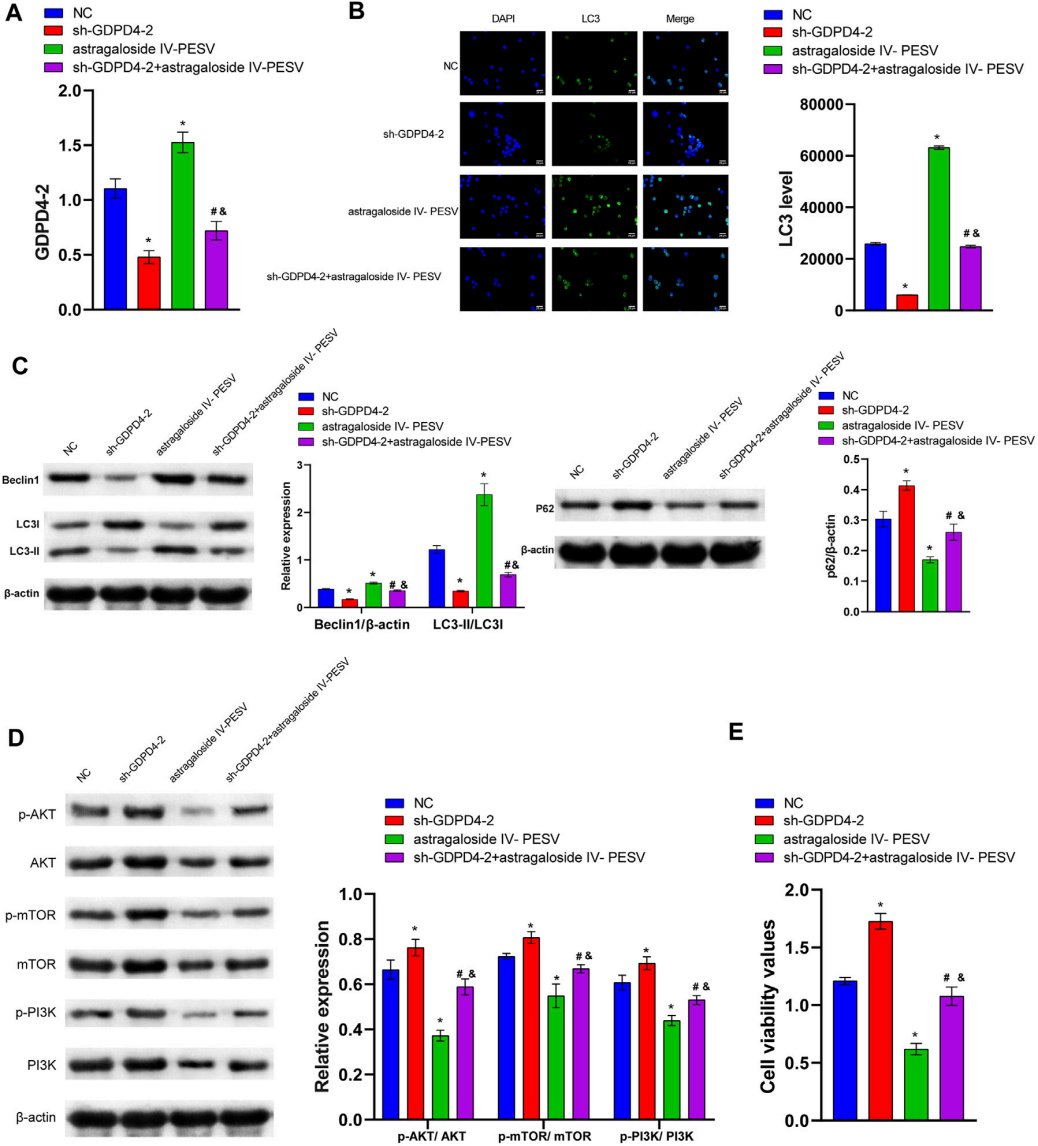
Astragaloside IV- PESV regulates PI3K/AKT/mTOR signaling via GDPD4-2. **(A)** The expression level of GDPD4-2. **(B)** LC3 and DAPI immunofluorescence staining were performed to detect autophagy. **(C)** The LC3, Beclin1, and P62 expression were determined by Western blot. **(D)** The protein expression of the PI3K/AKT/mTOR signaling pathway. **(E)** Cell activity was determined by the CCK8 assay. *p < 0.05 vs. NC group, #*p* < 0.05 vs. sh-GDPD4-2 group, &*p* < 0.05 vs. Astragaloside IV-PESV group.

Compared with the NC group, the expression of GDPD4-2 was significantly decreased in the sh-GDPD4-2 group and significantly increased in the Astragaloside IV-PESV group (Figure 5A). The expression of GDPD4-2 was significantly decreased in the sh-GDPD4-2 + Astragaloside IV-PESV group compared with that of the Astragaloside IV-PESV group (Figure 5A). Silencing GDPD4-2 inhibited the expression of LC3 and Beclin1 protein in LNCaP, and Astragaloside IV-PESV promoted the expression of LC3 and Beclin1 protein in LNCaP-silenced GDPD4-2 (Figures 5B,C). Silencing GDPD4-2 promoted the expression of P62 protein in LNCaP, while Astragaloside IV-PESV inhibited the expression of P62 protein in LNCaP-silenced GDPD4-2 (Figure 5C). Compared with the sh-GDPD4-2 group, the expression of p-PI3K, p-AKT, and p-mTOR proteins was decreased in the sh-GDPD4-2 + Astragaloside IV-PESV group (Figure 5D). Silencing GDPD4-2 promoted the proliferation of LNCaP, which was reversed by Astragaloside IV-PESV intervention (Figure 5E). These results demonstrated that Astragaloside IV-PESV regulated the PI3K/AKT/mTOR signaling pathway of LNCaP *via* GDPD4-2.

### *In Vivo* Experiments Demonstrated That Silencing GDPD4-2 Reversed the Inhibitory Effect of Astragaloside IV-PESV on PCa

**FIGURE 6:**
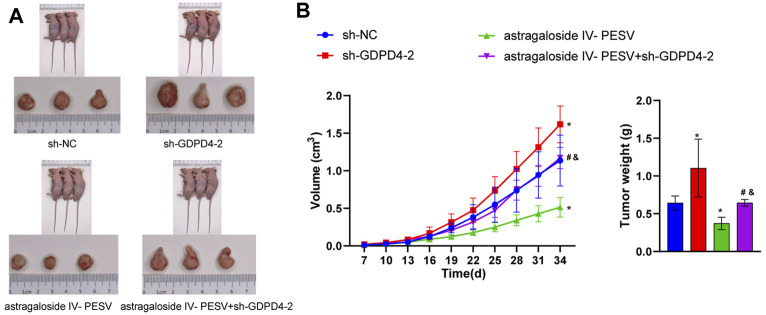
Down-regulation of GDPD4-2 reversed the therapeutic effect of Astragaloside IV-PESV on PCa. **(A)** The tumor tissue of mice was observed. **(B)** The tumor volume and tumor weight of mice were analyzed. *p < 0.05 vs. sh-NC group, #*p* < 0.05 vs. sh-GDPD4-2 group, &*p* < 0.05 vs. Astragaloside IV-PESV group.

Compared with the sh-NC group, the sh-GDPD4-2 group had an increased tumor volume (Figures 6A,B). Astragaloside IV-PESV inhibited tumor growth and volume compared with the sh-NC group (Figures 6A,B). Compared with the sh-NC group, the tumor weight of PCa mice was increased in the sh-GDPD4-2 group and decreased in the Astragaloside IV-PESV group (Figure 6B). Compared with the Astragaloside IV-PESV group, the tumor weight of PCa mice in the Astragaloside IV-PESV + sh-GDPD4-2 group was increased (Figure 6B). These results suggested that silencing GDPD4-2 reversed the inhibitory effect of Astragaloside IV-PESV on PCa.

## Discussions

Animal and botanical TCM are a rich source of gene modulators that can be used to prevent and treat cancer (Yang et al., 2014; Ma et al., 2015; Hnit et al., 2021; Hwang et al., 2021). Astragalus-containing TCM had a better anti-gastric cancer efficacy in patients with chemotherapy (Cheng et al., 2021). Astragalus active extracts, including triterpene saponins, flavonoids, polysaccharides, and other components, exert various effects such as antioxidant, anti-inflammatory, and anti-tumor by regulating autophagy (Shan et al., 2019). In particular, Astragaloside IV, as one of the main components of triterpene saponins, has been confirmed to be a key component of the Astragalus’ anticancer effect in breast, lung, gastric, and other cancers (Chen T. et al., 2021). However, studies on the effects of Astragaloside IV on PCa by regulating autophagy are largely lacking. In recent years, scorpion has been focused on scorpion venom in anti-tumor research (Dueñas-Cuellar et al., 2020). Scorpion venom consists of complex bioactive peptides, and PESV is extracted from scorpion venom. PESV has proven to be a promising anticancer drug (Mikaelian et al., 2020). PESV mediated the inhibition of hepatocellular carcinoma by up-regulating the NK cell activity (Chen et al., 2016a). PESV Smp43 can regulate the PI3K/AKT/mTOR pathway by inducing autophagy in liver cancer cells to exert an anti-tumor effect (Chai et al., 2021). Our study firstly confirmed that the A–S drug pair and their extracts (Astragaloside IV and PESV) promoted the expression of LC3 protein in cancer tissues and inhibited the expression of P62 and PI3K–AKT pathway proteins, and then inhibited the formation of PCa in mice. The above studies proved that the A–S drug pair extract could be used as a potential drug for the treatment of PCa, but the specific mechanism of action still needs to be further studied.

LncRNAs are involved in the development of various cancers including PCa. Some lncRNAs are overexpressed in PCa, and some are under-expressed (Misawa et al., 2017). We found that GDPD4-2, AC144450.1, LINC01513, AC004009.2, AL096869.1, AP005210.1, and BX119924.1 were significantly underexpressed in PCa. LINC01513 was underexpressed in nasopharyngeal carcinoma (Wang J. et al., 2021). However, AC144450.1 has been found to be highly expressed in the breast tissue relative to the adjacent tissue (Hassani et al., 2021). This is because different cancer types and causes may have different regulatory mechanisms (Xu et al., 2019). Astragaloside IV has been reported to inhibit liver cancer by regulating lncRNA ATB (Li et al., 2018). Similar to this report, our study found that Astragaloside IV might inhibit PCa by up-regulating GDPD4-2. Indeed, the regulatory function of PESV on lncRNAs has not been reported yet. We found that PESV up-regulates GDPD4-2, which is consistent with the multiple regulatory mechanism of lncRNA-PCa.

Silence of GDPD4-2 cannot completely block the effect of Astragaloside IV-PESV, suggesting that Astragaloside IV and PESV might affect PCa progress through other pathways. Astragaloside IV exerts anticancer effects, *via* the lncRNA TRHDE-AS1, in breast cancer (Hu et al., 2021). Astragaloside IV inhibits lung cancer progression through the MAPK pathway (Xu et al., 2018). Astragaloside IV has potential therapeutic effects on gastric cancer by regulating microRNA-195-5p (Liu et al., 2021). AaTs-1 (a PESV) inhibits glioblastoma proliferation *via* the p53 and FPRL-1 pathway (Aissaoui-Zid et al., 2021). Due to time and funding reasons, we did not conduct in-depth research on the above pathways. In future studies, we will continue to investigate whether Astragaloside IV and PESV affect cancer development by regulating other pathways.

Network pharmacology analysis (Bioinformatics tools) is an auxiliary tool for the study of the mechanism of TCM, which can easily and quickly screen out the active components and action targets of TCM compounds (Zhang et al., 2019). However, the application of network pharmacology analysis has many limitations (Luo et al., 2020; Silverman et al., 2020). In the current network pharmacology research, the chemical components found in the composition of the compound are taken as the research object, but the monomer of TCM is different from a chemical substance with a single chemical composition, and it is not a simple collection of a group of chemical components. A TCM monomer often contains multiple chemical components, and the same chemical component may come from different TCM monomers. The scorpion venom is mainly composed of lipids, organic acids, a small amount of free amino acids, and PESV (Yu et al., 2020). In addition, the research on scorpion is less than that on Astragalus, and the existing database of scorpion is incomplete. Therefore, for the A–S drug pair, network pharmacology analysis can assist in making some general predictions and summaries, and its real firm and exact effects still depend on a more accurate and comprehensive laboratory methodology.

A–S pair drugs include Astragalus and scorpion. Among them, the scorpion particles were derived from the whole body of *Buthus martensii Karsch* (BmK), a scorpionidae animal, including scorpion venom. PESV is a polypeptide extracted from scorpion venom. Scorpion is a TCM for animals with complex main active ingredients. San’ao decoction with scorpion has been shown to relieve asthma (Wang P. et al., 2021). It is well known that scorpion venom is the leading cause of human poisoning and even death by scorpion sting (So et al., 2021). Reports in recent years indicated that scorpion venom might be the main active ingredient in scorpion that exerts anticancer effects. For example, Androctonus amoreuxi scorpion venom significantly had cytotoxic and anti-proliferative effects on PCa cells (Akef et al., 2017). Polypeptide extracted from scorpion venom (PESV) induced growth inhibition of PCa cells (Zhang et al., 2009). Therefore, PESV was selected as our research object.

Wu et al. used bioinformatics tools to identify potential targets (PI3K/AKT pathway) for PCa from the main active components contained in scorpion (stearin, 20-hexadecanoylingenol, cholesterol, etc.) and Astragalus (bifendate, hederagenin, kaempferol, etc.) (Wu et al., 2021). However, many articles have confirmed the anticancer effects of Astragaloside IV and PESV by experimental methods (Chen et al., 2016a; Chen T. et al., 2021). The HPLC results confirmed that Astragaloside IV existed, and LC-MS/MS analysis also confirmed that scorpion venom polypeptide unique amino acid sequences, BmK_AGAP and BmK_AngM1, existed in scorpion. Therefore, different from the study by Wu et al., Astragaloside IV and PESV were selected as the focus of our subsequent cell and nude mouse tumorigenic studies. Our study confirmed that Astragaloside IV-PESV was the important active ingredient of A–S drug pair extracts for inhibiting PCa. Of course, there may be other active ingredients in the A–S drug pair, such as Astragalus polysaccharides (Bamodu et al., 2019) and Astragalus II (Wang M. et al., 2017), that have anticancer properties. Other components of the A–S pair have not been studied for reasons of funding and time. We plan to continue research further in a future research.

Network pharmacology and molecular docking techniques suggested that the A–S drug pair has the potential to treat PCa (Wu et al., 2021). However, no experimental studies on active ingredients and mechanisms have been conducted. Compared with previous studies (Wu et al., 2021), this study further investigated A–S, Astragalus, and scorpion, which might inhibit tumor development by activating autophagy and inhibiting the PI3K/AKT/mTOR pathway based on network pharmacology. There are many reports on the effects of lncRNAs on PCa, such as PCAT19 (Hua et al., 2018), PCAT6 (Lang et al., 2021), OIP5-AS1 (Zhang et al., 2021), etc. However, GDPD4-2 has never been reported to PCa. *In vitro* studies showed that Astragaloside IV combined with PESV might activate autophagy and inhibit the PI3K/AKT/mTOR pathway by promoting the expression of GDPD4-2. Finally, the tumor formation experiments in nude mice further confirmed that Astragaloside IV combined with PESV may inhibit tumor development through the GDPD4-2 pathway. In conclusion, this study clarified that the A–S drug pair inhibited the occurrence and development of PCa by regulating the GDPD4-2/PI3K/AKT/mTOR pathway and autophagy. This study provides a new reference idea for the study of the combined effect of TCM and also lays a theoretical foundation for the future combination of A–S drugs in the treatment of PCa. In conclusion, the study is very innovative and meaningful.

BmK_AngM1 was the main active component of the PESV extract (Wang QH. et al., 2017). However, BMK_AngM1 might not be the unique active component present in the PESV extract. For example, Chlorotoxin (CTX), HsTX1, and BmK AS have anticancer, analgesic, and antiepileptic effects, respectively (Li et al., 2019). The study found that BmK_AGAP was also presented in the scorpion. On the one hand, BmK_AngM1 and BmK_AGAP were reported to have significant analgesic effects (Wang QH. et al., 2017; Kampo et al., 2019). On the other hand, the antiproliferative effect of BmK_AGAP on the early stage of breast cancer has been reported (Doudou et al., 2019; Richard et al., 2020). Our study found that Astragaloside IV and PESV have anti-tumor effects in PCa. We speculated that BmK_AngM1 and BmK_AGAP might be the active components of PESV to inhibit PCa proliferation. The protein extracted from the scorpion was cut into small molecular peptides by enzymes and then scanned by MS, and the procedure was relatively complicated. The detection of small-molecule peptides with only 8–30 amino acids is considered the best. Therefore, there may be other small-molecule polypeptides in the scorpion that have not been detected. Due to funding and time constraints, the content of this section has not been studied. In the next study, we will further identify the main anticancer active small-molecule peptides in PESV.

The gradual transformation of LC3 from the cytosol (LC3 I) form to the membrane-bound lipidosis (LC3 II) form was used to measure the occurrence of autophagy. Theoretically, in the representative image of immunofluorescence, LC3 II will aggregate to the autophagosome membrane and appear as bright spots, and LC3 II will diffuse (Kabeya et al., 2000; Chen et al., 2019). However, in many cases, the separation of LC3 I and LC3 II in immunofluorescence is not significant (Cao et al., 2019; Rong et al., 2020). In our cell experiments, some bright spots could be observed on the cells, suggesting that LC3 I might be transformed into LC3 II after Astragaloside IV and PESV treatment. After further experiments, the results of western blot showed that the A–S drug pair and its active components (Astragaloside IV and PESV) promoted the expression of LC3 II and decreased the expression of LC3 I. In addition, the changes of autophagy-related proteins (Becline 1 and P62) further illustrated the effects of A–S drugs on autophagy. The above results illustrated that the A–S drug pair and its active components (Astragaloside IV and PESV) promoted autophagy.

Rapamycin is a macrolide that is originally developed as an antifungal agent (Jiang et al., 2021). Rapamycin is useful in the treatment of various cancers due to its inhibitory effect on the PI3K/AKT/mTOR pathway (Alzahrani, 2019). Both AKT and mTOR are downstream targets of PI3K, which stimulate protein synthesis, cell growth, and proliferation. mTOR is an essential component of this network and a PI3K-related serine–threonine kinase (Alzahrani, 2019). mTOR regulates the balance of immune responses by AKT phosphorylation (Cook et al., 2020). At present, the research on rapamycin focuses on the research on the PI3K/AKT/mTOR pathway as a whole, while the research on the individual PI3K/AKT and its phosphorylation is less. Additionally, we found that rapamycin inhibited retinoblastoma cell proliferation by inhibiting the PI3K/AKT protein expression (Yao et al., 2020). In renal failure studies, rapamycin has been shown to inhibit the expression of p-PI3K/PI3K and p-AKT/AKT (Jia et al., 2022). Similar to the above studies, our study found that rapamycin could inhibit the expression of p-PI3K/PI3K and p-AKT/AKT in PCa cells. Therefore, we speculated that there might be a negative feedback regulation mechanism in the PI3K/AKT/mTOR pathway. Rapamycin might inhibit mTOR, which, in turn, inhibit AKT and finally inhibit the PI3K pathway. However, the specific mechanism is not clear. In follow-up studies, it is necessary to further explore the deeper regulatory mechanism of the PI3K/AKT pathway from *in vivo* and *in vitro* experiments.

Considering the overall consistency of the entire study, the LNCaP cell line was used for both *in vitro* and *in vivo* experiments. Due to funding and time constraints, we were unable to provide additional cell lines for this comparison and other experimental studies. In future studies, more cell lines will be further investigated. In addition, autophagy and apoptosis often accompany each other in the process of regulating tumor cells. Dietary phytochemicals regulate autophagy and apoptosis in cancer (Patra et al., 2021). TCM may exert anticancer effects by regulating autophagy and apoptosis (Wang K. et al., 2021). Due to time and funding constraints, we have not studied it for the time being. In a future research, we will investigate further.

## Conclusion

Therefore, the above study proved that Astragaloside IV-PESV is the pharmacodynamic component of A–S against PCa, and its mechanism may be related to the regulation of the GDPD4-2/PI3K/AKT/mTOR pathway and autophagy. This study preliminarily elucidates the potential mechanism of action of the A–S drug pair in the treatment of PCa by Astragaloside IV-PESV, with a view to developing new therapeutic strategies for PCa.

## Data Availability

The original contributions presented in the study are included in the article/Supplementary Material; further inquiries can be directed to the corresponding authors.

## Supplementary Material

Supplementary Figure S1 The chemical structures of various screened active ingredient monomers in the A–S drug pair. **(A)** The chemical structure of the active ingredient monomers in the Astragalus. **(B)** The chemical structure of the active ingredient monomers in the scorpion.
