# Corrigendum: Astragalus–Scorpion drug pair inhibits the development of prostate cancer by regulating GDPD4-2/PI3K/AKT/mTOR pathway and autophagy

**DOI:** 10.3389/fphar.2025.1417603

**Published:** 2025-03-20

**Authors:** Xujun You, Yongrong Wu, Qixin Li, Wen Sheng, Qing Zhou, Wei Fu

**Affiliations:** ^1^ Graduate School of Hunan University of Chinese Medicine, Changsha, China; ^2^ Department of Andrology, Shenzhen Bao’an Traditional Chinese Medicine Hospital, Guangzhou University of Chinese Medicine, Shenzhen, China; ^3^ College of Integrated Traditional Chinese and Western Medicine, Hunan University of Chinese Medicine, Changsha, China; ^4^ Andrology Laboratory, Hunan University of Chinese Medicine, Changsha, China; ^5^ Department of Andrology, The First Affiliated Hospital of Hunan University of Chinese Medicine, Changsha, China

**Keywords:** Astragalus–Scorpio, prostate cancer, PI3K/AKT, Astragaloside IV, polypeptide extract from scorpion venom, autophagy

In the published article, there was an error in the legend for **Figure 4** as published. The original description has ambiguity, which may cause readers to misunderstand. The corrected legend appears below.

FIGURE 4 | In PCa tissues and LNCaP cells, GDPD4-2 expression was decreased compared to non-tumorigenic prostate epithelial cells but increased following treatment with the herb pair Astragalus IV and PESV. **(A)** Volcano plot showed the lncRNA expression. **(B)** The differential lncRNA expression in RWPE-1 and LNCaP cells, *p < 0.05 vs. RWPE-1 group. **(C)** The differential lncRNA expression was analyzed by RT-qPCR in LNCaP cells after treatment with Astragaloside IV-PESV. *p < 0.05 vs. control group, #*p* < 0.05 vs. Astragaloside IV group, &*p* < 0.05 vs. PESV group.

In the published article, there was an error in the legend for **Figure 5** as published. The original description was not consistent with its results. The corrected legend appears below.

FIGURE 5 | Astragaloside IV- PESV regulates PI3K/AKT/mTOR signaling via GDPD4-2. **(A)** The expression level of GDPD4-2. **(B)** LC3 and DAPI immunofluorescence staining were performed to detect autophagy. **(C)** The LC3, Beclin1, and P62 expression were determined by Western blot. **(D)** The protein expression of the PI3K/AKT/mTOR signaling pathway. **(E)** Cell activity was determined by the CCK8 assay. *p < 0.05 vs. NC group, #*p* < 0.05 vs. sh-GDPD4-2 group, &*p* < 0.05 vs. Astragaloside IV-PESV group.

In the published article, there was an error in Figure 3 as published. The WB blot image of p-PI3K was mistakenly added in place of p-AKT and PI3K. The corrected Figure 3 and its caption appear below.

**FIGURE 3:**
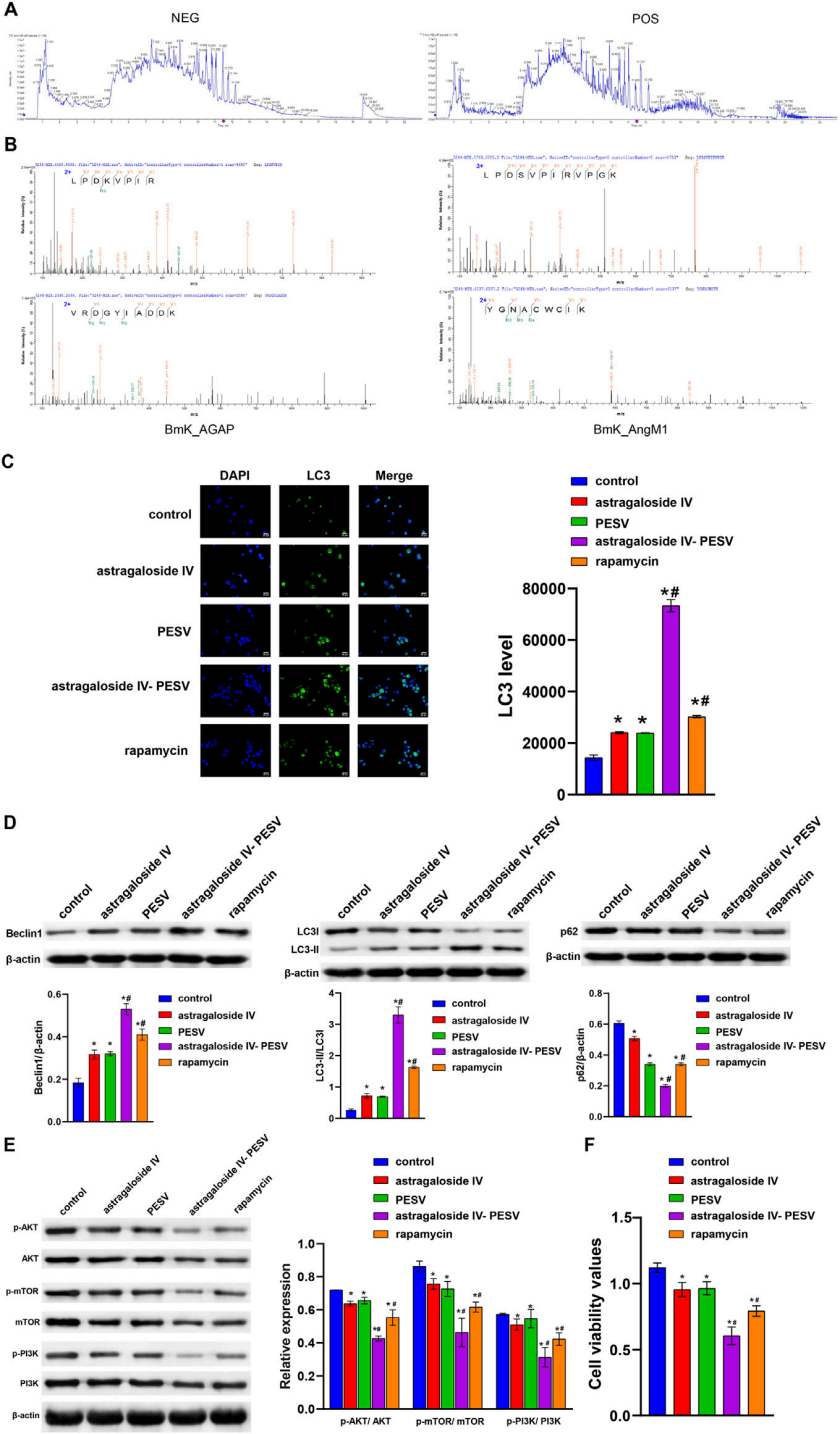
The main bio-active components in the herb pair A–S suppressed LNCaP cell proliferation and migration through the PI3K/AKT/mTOR pathway. **(A)** LC–MS/MS chromatogram of the bio-active components in Astragalus. **(B)** The LC-MS/MS analysis of PESV in scorpion. **(C)** LC3 and DAPI immunofluorescence staining were performed to detect autophagy. **(D)** The LC3, Beclin1, and P62 expression were determined by Western blot. **(E)** The protein expression of the PI3K/AKT/mTOR signaling pathway. **(F)** Cell activity was determined by the CCK8 assay. *p < 0.05 vs. control group, #*p* < 0.05 vs. Astragaloside IV or PESV groups.

The authors apologize for these errors and state that this does not change the scientific conclusions of the article in any way. The original article has been updated.

