# Supplementary figures and images for: Astragalus–Scorpion Drug Pair Inhibits the Development of Prostate Cancer by Regulating GDPD4-2/PI3K/AKT/mTOR Pathway and Autophagy
Source: Front Pharmacol. 2022 Jun 29;13:895696. doi: 10.3389/fphar.2022.895696 (PMC9277392; doi:10.3389/fphar.2022.895696)

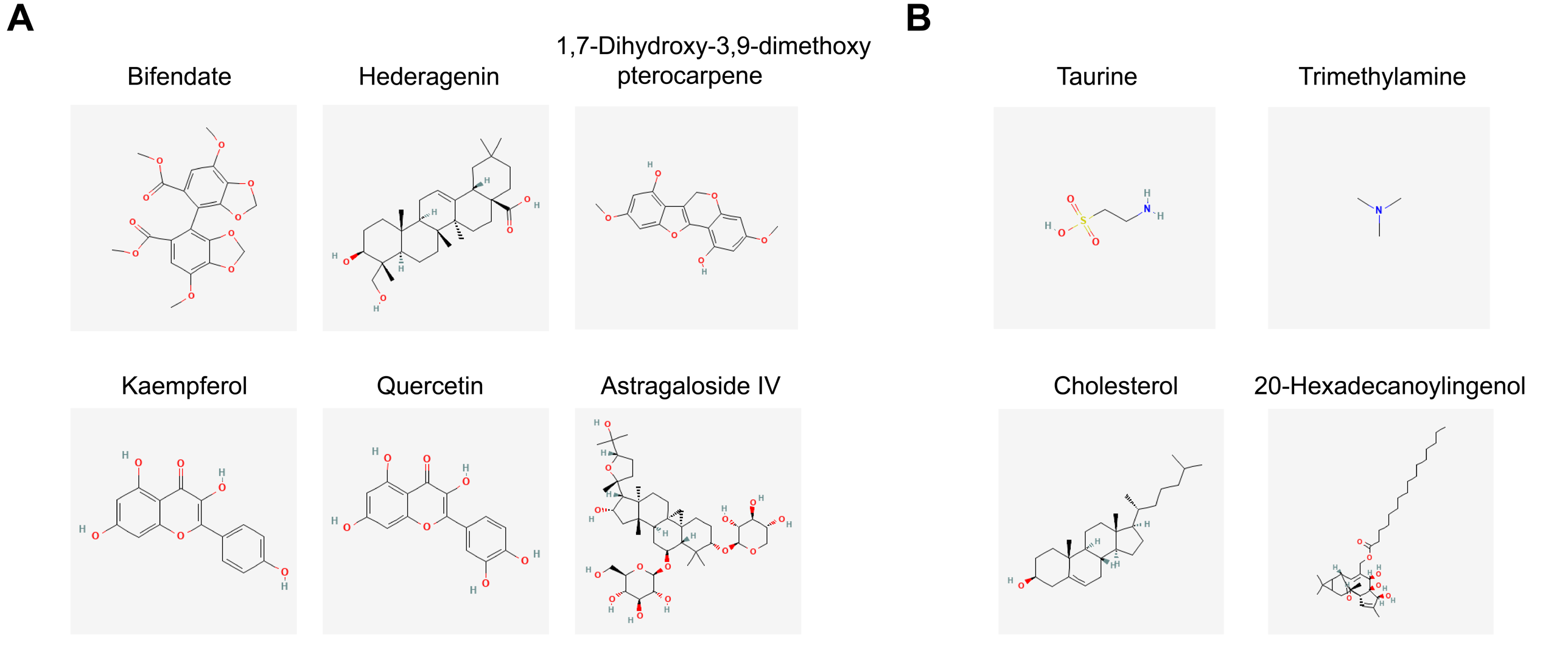

Supplement: Supplementary file 1 [file Image1.JPEG]
